# WRKY10 Regulates Seed Size through the miR397a-LAC2 Module in *Arabidopsis thaliana*

**DOI:** 10.3390/genes15081040

**Published:** 2024-08-07

**Authors:** Wenbin Guo, Ke Yang, Hang Ye, Jialing Yao, Jing Li

**Affiliations:** 1 College of Life Science and Technology, Huazhong Agricultural University, Wuhan 430070, China; 2 Sanya Institute of Breeding and Multiplication, Hainan University, Sanya 572025, China; 3 School of Tropical Agriculture and Forestry, Hainan University, Haikou 570228, China; 4 Hainan Yazhou Bay Seed Laboratory, Sanya 572025, China

**Keywords:** seed size, endosperm, IKU, *miR397a*, LACCASE

## Abstract

In angiosperms, seed size is a critical trait that is influenced by the complex interplay between the endosperm and seed coat. The *HAIKU* (*IKU*) pathway, involving the transcription factor WRKY10, plays a crucial role in regulating seed size in *Arabidopsis thaliana*. However, the downstream targets of WRKY10 and their roles in seed size determination remain largely unexplored. Here, we identified *LACCASE2* (*LAC2*), a laccase gene involved in lignin biosynthesis, as a new downstream target of WRKY10. We observed that the expression of *LAC2* was upregulated in the *mini3* mutant, which is defective in WRKY10. We demonstrated that WRKY10 directly binds to the promoter of miR397a, activating its expression. miR397a, in turn, represses the expression of *LAC2*. Genetic analyses revealed that a mutation in *LAC2* or overexpression of miR397a partially rescued the small seed phenotype of the *MINISEED3* (*MINI3*) mutant *mini3*. Conversely, the overexpression of *LAC2* in the wild type led to a decrease in seed size. These findings suggest that *LAC2* functions as a negative regulator of seed size, and its expression is modulated by WRKY10 through miR397a. Our study uncovers a novel WRKY10-miR397a-LAC2 pathway that regulates seed size in *Arabidopsis*, providing new insights into the complex regulatory network governing seed development in plants.

## 1. Introduction

Seed size is a critical trait in plants, as it directly influences seed yield, seedling vigor, and overall reproductive success. After the gametophyte develops and matures, angiosperms undergo a double fertilization process. Double fertilization refers to the fertilization of two male sperm cells on two female gametes (egg cell and central cell) in angiosperms, leading to the development of the embryo and endosperm. The endosperm is a nutrient tissue that supports embryo growth. It is surrounded by the seed coat, which originates from the maternal integument. After double fertilization, a diploid embryo with a genome ratio of (1m:1p) and a triploid endosperm with a genome ratio of (2m:1p) are formed. Although the embryo is the only component that forms the next generation, studies suggest that its contribution to seed growth and development may be limited [1]. Instead, the cooperation between the endosperm and seed coat plays a crucial role in determining the final seed size [2,3].

In *A. thaliana*, the IKU pathway has emerged as a key regulator of endosperm growth and seed size determination [2]. This pathway involves three key components: the VQ domain protein IKU1, the LRR receptor kinase IKU2, and the transcription factor WRKY10, which is encoded by *MINI3* [2,4,5,6]. The *mini3* homozygous mutant produced seeds that are significantly smaller in both weight and size than the wild type, which is associated with precocious endosperm cellularization [5]. While the IKU pathway has been shown to regulate early endosperm growth through the cytokinin signaling pathway, with the downstream target *CYTOKININ OXIDASE2* (*CKX2*) partially mediating this effect [7], the involvement of additional downstream targets remains largely unexplored.

Lignin, a complex polymer derived from phenylalanine, plays a vital role in the structure and function of the plant cell wall [8,9]. Its biosynthesis involves the generation of monolignols in the cytoplasm, their transport to the cell wall, and subsequent polymerization by enzymes such as laccases (LACs) and peroxidases (PRXs) [10,11,12,13]. In rice, the interaction between *OsLAC* (LOC_Os05g38420) and OsmiR397 has been reported to be involved in the regulation of seed size [14]. The overexpression of OsmiR397 in rice significantly enhances grain size and weight, ultimately leading to enhanced grain yield [14]. This enhancement is attributed to the downregulation of *OsLAC.* Moreover, the overexpression of *OsLAC* genes leads to small seeds compared to those of wild-type plants [14]. In *Arabidopsis*, there are two members of 21 nt miR397 with only one base variance in their mature sequences, miR397a (ucauugagugcaGcguugaug) and miR397b (ucauugagugcaUcguugaug), encoded by AT4G05105 and AT4G13555, respectively [15]. Both miR397 genes exhibit similar spatial and temporal expression patterns in a majority of tissues, with higher levels observed in seeds and siliques compared to rosette leaves and flowers [16]. In *Arabidopsis*, *LAC2* has been identified as a target of miR397 and is known to regulate lignin deposition in roots [17,18,19,20]. However, the potential role of *LAC2* and its regulation by miR397 in endosperm growth and size determination have not been investigated.

In this study, we uncovered a novel role for the miR397a-LAC2 module in regulating seed size in *Arabidopsis*, downstream of the IKU pathway component WRKY10. We observed that the expression of *LAC2* was upregulated in the *mini3* mutant, which is defective in WRKY10. Through a combination of genetic, biochemical, and molecular approaches, we demonstrate that WRKY10 directly activates the expression of miR397a, which in turn represses *LAC2* expression. Our findings reveal that *LAC2* acts as a negative regulator of seed size and that the WRKY10-miR397a-LAC2 pathway represents a novel regulatory mechanism governing seed development in *Arabidopsis*. This study not only expands our understanding of the complex network regulating seed size but also highlights the potential for manipulating this pathway to improve seed traits in crops.

## 2. Materials and Methods

### 2.1. Plant Materials and Growth Conditions

*A. thaliana* ecotype Columbia (Col-0) and *Nicotiana benthamiana* are used in this study. The *mini3* (SM_3_33099) line was obtained from the Arabidopsis Biological Resource Center (ABRC: https://abrc.osu.edu/, accessed on 25 July 2022). The *lac2* (SALK_025690) line was obtained from the AraShare (https://www.arashare.cn/index/, accessed on 8 June 2023). All plants were grown in a greenhouse at 22 °C with 60% humidity under a long day condition (16 h light/8 h dark).

### 2.2. Plasmid Construction

To construct vectors for *pFWA* (*FLOWERING WAGENINGEN*)*::LAC2*, 3666 bp (from −3666 to −1) of *FWA* promoters and *LAC2* coding sequences were amplified by PCR using Phanta Max Super-Fidelity DNA Polymerase (Vazyme, Nanjing, China) from genomic DNA and cDNA and cloned into *pSR100* binary vectors using Hieff Clone^®^ Plus One Step Cloning Kit (YEASEN, Shanghai, China), which allows for the selection of transgenic seeds via mCherry expression driven by a seed specific promoter, *At2S3* [21]. To construct vectors for *pFWA::miR397a*, 3666 bp (from −3666 to −1) of *FWA* promoters and the genomic DNA sequences of the pre-miR397a were cloned into the *pSR100* binary vector. The WRKY10 coding sequence was amplified and cloned into pMAL-c2x for expression in *Escherichia coli* BL21(DE3).

For the dual-luciferase assay, a 750 bp promoter of miR397a (from −750 to −1) was amplified from genomic DNA and cloned into a pGreenII_0800 vector. And the coding sequences of WRKY10 were amplified and cloned into pFGC5941 driven by the 35S promoter.

### 2.3. Dual-Luciferase Assay

Dual-luciferase assay-related plasmids were co-transformed into *Agrobacterium tumefaciens* strain GV3101 with plasmid *pSoup*. After being incubated to OD_600_ = 1.0, the *A. tumefaciens* GV3101 cells were collected by centrifugation at 1000× *g* for 5 min, followed by being resuspended by a stock buffer containing 10 mM MES, pH = 5.7; 10 mM MgCl_2_; and 100 μM acetosyringone. Equal volumes of two cultures with 35S::WRKY10 and LUC constructs were mixed prior to injection into the *N. benthamiana* leaves. Three days after injection, luciferase activities were measured using Dual-Luciferase Reporter Assay System (Promega, Madison, WI, USA).

### 2.4. Protein Expression and Purification

After being incubated at 37 °C with shaking at 200 rpm until the OD_600_ reached 0.8, cells containing a plasmid were treated by 0.5 mM isopropyl-β-dthiogalactopyranoside (IPTG) at 16 °C for 12 h for the expression of MBP-WRKY10 protein. The well-centrifugated and supernatanted cells were lysed in a lysis buffer with 25 mM Tris-HCl, pH = 7.5; 20 mM NaCl; 1 mM Na_2_-EDTA; 10 mM 2-mercaptoethanol; and 1 mM PMSF by a low-temperature ultra-high-pressure cell disrupter (JN-10C, JNBIO, Guangzhou, China). The supernatant was passed through amylose resin (New England Biolabs, Ipswich, MA, USA), after centrifugation at 12,000 rpm for 50 min at 4 °C. The protein was eluted by the lysis buffer with 10 mM maltose, which was further purified by ion exchange chromatography HiTrap Q FF followed by Superdex 200 Increase 10/300 GL (GE Healthcare, Chicago, IL, USA) using buffer C containing 25 mM Tris-HCl, pH = 8.0, and 250 mM NaCl. The peak fractions were collected for a further EMSA assay.

### 2.5. Electrophoretic Mobility Shift Assay

For the electrophoretic mobility shift assay, 5′6-CarBoxyflfluorescein (FAM)-labeled probes and unlabeled competitor probes with W-Box in the promoter of the WRKY10 target gene were selected and annealed with equimolar amounts of complementary unlabeled probes. Proteins were incubated with a FAM-labeled probe for 15 min at 22 °C in the final solution containing 25 mM Tris-HCl, pH = 8.0; 5 mM MgCl_2_; 150 mM KCl_2_; 1 mM DTT; 60 μg/mL salmon sperm DNA; and 2.5% (*v*/*v*) glycerol. The reaction products were then resolved on 6% (*v*/*v*) native acrylamide gels (29:1 acrylamide/bis-acrylamide) in 0.5× Tris–Boric acid buffer under an electric field of 120 V for 1 h. Gels were visualized using the tool Amersham Typhoon5 (GE Healthcare, Chicago, IL, USA).

### 2.6. RNA Isolation and qRT-PCR

To analyze gene expression in seeds, we performed emasculation on flowers at the 12c stage [22], waited 24 h for pollination, self-pollinated the flowers, and then waited for an additional 72 h. Following this, 15 siliques without valves from a single plant were collected for the purpose of RNA isolation. Total RNA was extracted using RNAprep pure Plant Kit (TIANGEN, Beijing, China). First-strand cDNA was generated via a reverse transcription reaction with a HiScript^®^II 1st Strand cDNA Synthesis kit (Vazyme, China). The abundance of target genes and the internal control gene *ACTIN11* transcripts were detected using qRT-PCR with ChamQ Universal SYBR qPCR Master Mix (Vazyme, China) and three replicates were performed. The relative transcript abundance was calculated using the 2^−∆∆CT^ method. The numbers are presented as means ± SD from three replicates.

### 2.7. Seed Size Measurement

For the seed size analysis, seeds originating from plants grown under identical conditions were utilized. To capture high-resolution images of the mature seeds, a stereomicroscope (Nikon, Tokyo, Japan) was employed. The seeds were carefully spread out onto a plain white paper, ensuring that they were evenly distributed and not overlapping. Following image capture, the seed area was analyzed using ImageJ software [23].

## 3. Results

### 3.1. The LAC2 Is Upregulated in mini3 Seeds

**Figure 1:**
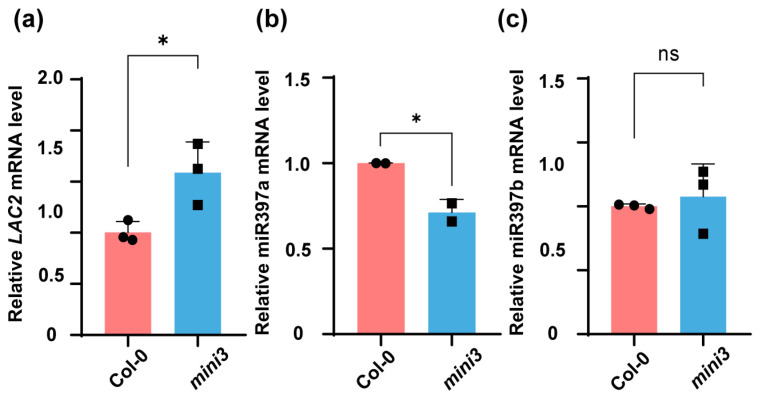
The expression of *LAC2*, miR397a, and miR397b in Col-0 and *mini3* 3 DAP seeds. (**a**–**c**) The relative expression of *LAC2* (**a**), miR397a (**b**), and miR397b (**c**) in wild-type Col-0 and *mini3*. The samples were collected at 3 days after pollination (3 DAP). The qPCR results were applied to three biological replicates, each with three technical replicates. The *ACTIN11* was used as an internal control. Data are presented as means ± SDs. The statistical significance was analyzed using Student’s *t*-test. * *p* < 0.05; ns indicates no change. Black circle and box shows the individual data points.

In rice, the interaction between *OsLAC* and OsmiR397 has been reported to be involved in the regulation of seed size [14]. In *Arabidopsis*, *LAC2* has been identified as a target of miR397 and is known to regulate lignin deposition in roots [17,18,19,20]. To investigate the potential genetic relationship between *LAC2* and miR397 in influencing seed size in *A. thaliana*, we assessed the expression of *LAC2* and miR397 in both the wild type and *mini3* mutant. A total of 15 siliques without valves from a single plant were collected for the isolation of total RNA. The RT-qPCR analysis revealed that the laccase gene *LAC2* was upregulated in the *mini3* mutants (Figure 1a). Concurrently, there was a significant reduction in the expression of miR397a in the *mini3* mutants (Figure 1b). In contrast, the expression level of miR397b remained unchanged (Figure 1c).

### 3.2. WRKY10 Directly Regulates the Expression of miR397a

It is known that the expression of *LACs* could be repressed by miRNAs, with *LAC2* being one of the targets of miR397 in *A. thaliana* [17,18,19,20]. Here, we noted that the expression of *LAC2* was upregulated in the *mini3* mutants. WRKY10 has been previously reported as a transcriptional activator involved in the expression of *CKX2*, *IKU2*, and its own gene [5,7]. The W-Box is a cis-regulatory element sequence, (T)TGAC(C/T), which is recognized by WRKY transcription factors. We checked the W-Box elements in the promoter region of *LAC2* and found that there was no TTGACY W-Box element on the 2000bp promoter. It seems improbable that WRKY10 directly represses *LAC2*. Based on these observations, our hypothesis is that WRKY10 may indirectly regulate *LAC2* by activating the expression of *miR397a*, which in turn would lead to the repression of *LAC2*.

**Figure 2:**
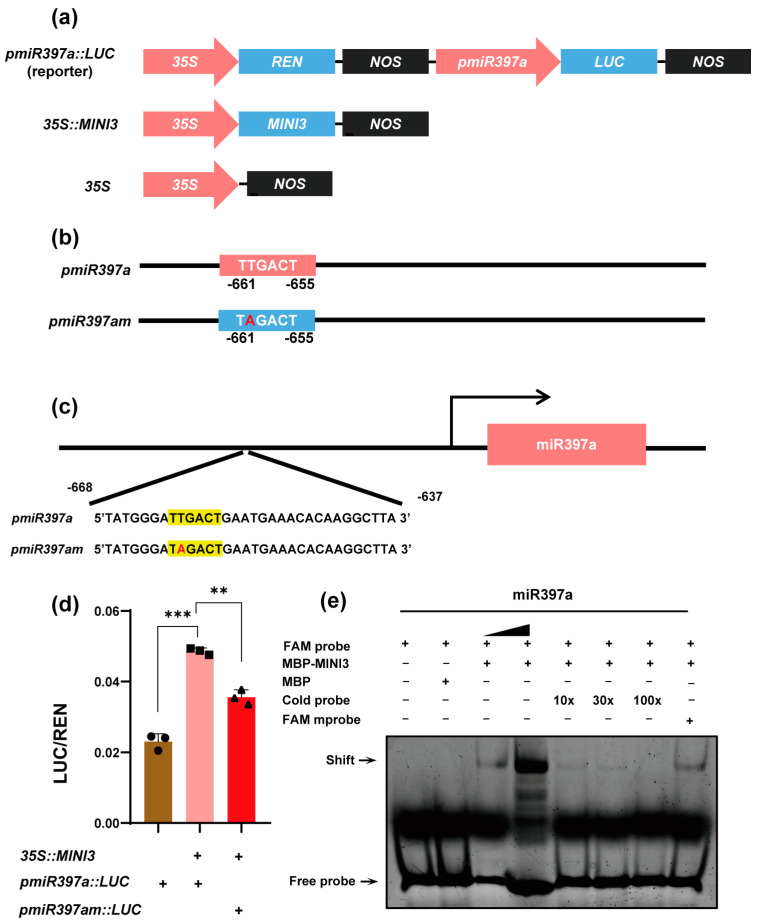
WRKY10 directly regulates the expression of miR397a. (**a**) A diagram of the *35S::MINI3*, *pmiR397a::LUC*, and *35S* empty constructs used for dual-luciferase reporter assays. (**b**) A diagram of the miR397a promoter used for dual-luciferase reporter assays. The W-Box elements and artificial mutation sites are labeled. (**c**) A diagram of the probe and mutated probe of miR397a that were used for EMSA assays. The yellow shaded and red part represent the W-Box sequence and mutation site of the mutated probes *pmiR397am*. (**d**) The dual-luciferase assay was used to detect the activation of miR397a by WRKY10. Relative LUC activities were calculated using values obtained from *N. benthamiana* leaves co-transformed with the specified reporter and effector plasmids. The normalized relative LUC activities (LUC/REN) are shown using three technical replicates and two biological replicates. Data are presented as means ± SDs. The statistical significance was analyzed using Student’s *t*-test. ** *p* < 0.01, *** *p* < 0.001. Black circle, box and triangle shows the individual data points. (**e**) The electrophoretic mobility shift assay (EMSA) result of WRKY10 binding to the miR397a promoter. The sequence and position of the FAM probe and FAM mprobe are indicated in Figure 2a. EMSA showed that WRKY10 protein could specifically bind to probe pmiR397a. The mobility shift was abolished by the addition of cold unlabeled probes, and no competitive activity was detected when the mutated probe was used. The triangle indicates that increasing amounts of the unlabeled promoter were used as a specific competitor of the DNA–protein interaction.

To determine whether WRKY10 can activate miR397a expression, we performed a dual-luciferase assay to examine the promoter activity of miR397a in response to WRKY10. We constructed transcriptional reporter plasmids with site-directed mutated W-Box elements on the miR397a promoter (Figure 2a,b). The promoter of miR397a harbors a canonical W-Box motif, known to be a binding site for WRKY transcription factors. In *N. benthamiana* leaves, the co-expression of WRKY10 led to a significant upregulation of *luciferase* (*LUC*) gene expression compared to the negative control (Figure 2d), indicating that WRKY10 can robustly enhance miR397a promoter activity. However, the mutation of the W-Box core sequence from TTGACT to TAGACT resulted in a reduced ability of WRKY10 to activate the *LUC* gene (Figure 2d). These findings highlight the critical role of the intact W-Box in the full activation of the miR397a promoter by WRKY10 and demonstrate that WRKY10 directly regulates miR397a expression via this specific cis-regulatory element.

To further validate the direct and specific interaction of WRKY10 with the W-Box element in the miR397a promoter, we performed electrophoretic mobility shift assays (EMSAs). The probes for these assays were designed from a region containing the W-Box element, located approximately 660 base pairs upstream of the ATG transcriptional start site (Figure 2c). EMSA results showed a distinct shift indicating the binding of recombinant WRKY10 to the FAM-labeled probe (Figure 2e). Notably, in lane 4, as we increased the concentration of the 5-fold FAM-labeled probe compared to lane 3, the intensity of the WRKY10-FAM probe complex also increased (Figure 2e). Subsequently, when we introduced a 100-fold excess unlabeled cold probe, it successfully competed with the FAM-labeled probe for WRKY10 binding (Figure 2e), resulting in a release of the labeled probe and confirming the specificity of the interaction. In contrast, a 100-fold excess mutated version of the probe, with alteration in the W-Box sequence, failed to compete for WRKY10 binding. These EMSA results provide strong evidence that WRKY10 binds directly and specifically to the W-Box element within the miR397a promoter.

### 3.3. LAC2 and miR397a Act Downstream of MINI3 and Are Involved in Seed Size Regulation

**Figure 3:**
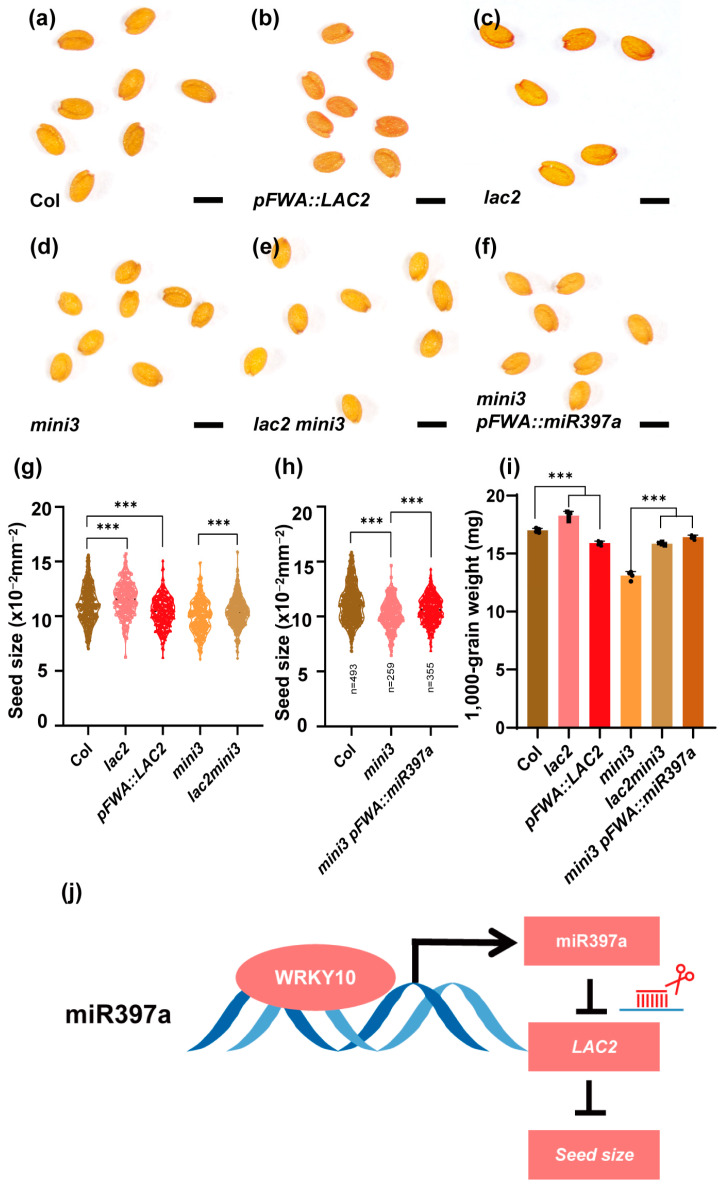
*LAC2* and miR397a act downstream of WRKY10 to regulate seed size. (**a**–**f**) Seed morphology of the wild type, *lac2*, *mini3*, *lac2mini3*, *pFWA::LAC2*, and *mini3 pFWA::miR397a*. Scale bar = 100 μm. (**g**) Seed size of the wild type, *lac2*, *mini3*, *lac2mini3*, and the transgenic plant *pFWA::LAC2*. n, number of seeds examined. (**h**) Seed size of the wild type, *mini3*, and the transgenic plant *mini3 pFWA::miR397a*. n, number of seeds examined. (**i**) The 1000-grain weight of the wild type, *lac2*, *mini3*, *lac2mini3*, *pFWA::LAC2*, and *mini3 pFWA::miR397a*. *n* = 4 replicates. Black circle, box and triangle shows the individual data points. (**j**) A model of seed size regulation by the WRKY10-miR397a-LAC2 pathway in *Arabidopsis*. The statistical significance was analyzed using Student’s *t*-test. *** *p* < 0.001. Data are presented as means ± SDs.

Given that *LAC2* exhibited an upregulated expression in the *mini3* mutant and WRKY10 directly binds to the miR397a promoter, we investigated the role of miR397a and *LAC2* in seed size determination. We proposed that the mutation of *LAC2* and overexpression of miR397a might reverse the small seed phenotype observed in *mini3*. To test this, a double-mutant line, *lac2mini3*, was created, along with an overexpression line for miR397a, using the endosperm-specific promoter *FWA* in the *mini3* background. Mature seed size measurements indicated a significant increase in seed size in both the *lac2mini3* double mutant (Figure 3e,g) and *mini3 pFWA::miR397a* overexpression line (Figure 3f,h) compared to the *mini3* mutant alone (Figure 3d,g). We further assessed the 1000-grain weight and noticed a significant enhancement in both l*ac2mini3* and *mini3 pFWA::miR397a* compared to the *mini3* variant (Figure 3i). These results suggest that the mutation of *LAC2* and the overexpression of miR397a could partially compensate for the *mini3* seed size phenotype.

Moreover, we speculated that the overexpression of *LAC2* could lead to smaller seeds in a wild-type background. In line with this, the *LAC2* overexpression line, under the control of the *FWA* promoter, showed a slight decrease in seed size compared to the wild type, which contrasts with the *lac2* mutant phenotype (Figure 3g). These results collectively support the hypothesis that *LAC2* and miR397a are involved in regulating seed size, functioning downstream of the *MINI3* gene.

## 4. Discussion

In this study, we have unveiled a novel regulatory pathway involving WRKY10, miR397a, and LAC2 in the control of seed size in *A. thaliana*. Our findings demonstrate that WRKY10 directly activates the expression of miR397a, which in turn represses the expression of *LAC2* (Figure 3j), a laccase gene involved in lignin biosynthesis. Interestingly, the miR397-LAC module has been previously implicated in various biological processes, such as flowering, seed development, and response to heavy metal stress in rice and other crops [14,24]. Our study extends the functional repertoire of this module by revealing its role in endosperm development and seed size regulation in *Arabidopsis*.

The identification of *LAC2* as a negative regulator of seed size provides new insights into the complex regulatory network governing seed development. The precocious cellularization of the endosperm in *iku* mutants [2] hints at the involvement of the IKU pathway in cell wall dynamics during the syncytial phase of endosperm development. The miR397a-LAC2 module, as a downstream component of the IKU pathway, may play a role in this process by modulating lignin deposition and cell wall properties. It is interesting to know why *lac2* knockdown increases the median seed size independent of *MINI3* and why *LAC2* overexpression may lead to reduced seed size. In rice, the interaction between *OsLAC* and OsmiR397 has been reported to be involved in the regulation of seed size [14]. The overexpression of OsmiR397 in rice transgenic lines significantly enhances grain size and weight. This improvement in grain yield is attributed to the downregulation of *OsLAC*, whose encoded laccase-like protein is involved in plant sensitivity to brassinosteroids, resulting in smaller grains in *OXLAC* lines compared to wild-type plants [14]. However, the exact biochemical functions of miR397a and *LAC2* in endosperm growth and their mechanistic link to seed size determination remain to be elucidated. Future studies should focus on investigating the spatiotemporal dynamics of lignin deposition in the endosperm cellularization and its potential impact on cell wall formation, nutrient transport, and seed filling.

While our study provides compelling evidence for the role of the miR397a-LAC2 module in seed size regulation in *Arabidopsis*, it is important to acknowledge the limitations of our work. We have focused on a single miR397 family member and one of its target genes, but the miR397 family is known to regulate multiple *LAC* genes [16,25,26]. To fully understand the regulatory network downstream of WRKY10, it will be necessary to investigate the roles of other miR397 family members and their respective *LAC* targets in seed development. Although the expression of miR397b in *A. thaliana* seeds is not regulated by WRKY10, it has been previously reported that there is also high expression in seeds [16]. Furthermore, it has also been reported that miR397b can directly regulate the expression of *LAC2* in roots, indicating that miR397b in seeds may also regulate the expression of *LAC2*, but not through the WRKY10 pathway. Additionally, our study was conducted in *Arabidopsis*, a model plant with relatively small seeds. To establish the conserved role of the miR397-LAC module in seed size regulation across diverse plant species, future research should explore the function of this module in crops with larger seeds, such as rice, maize, and legumes.

The identification of the WRKY10-miR397a-LAC2 pathway not only advances our understanding of seed size regulation but also highlights the avenues for crop improvement. Seed size is a key determinant of seed yield and quality, and manipulating this trait could have significant implications for agricultural productivity. Our findings suggest that modulating the expression of miR397a or its target *LAC* genes could be a potential strategy to enhance seed size in crops. However, before such approaches can be implemented, it will be crucial to assess the effects of these manipulations on other aspects of plant growth and development, as well as on seed quality and nutritional value.

In conclusion, our study uncovers a novel regulatory pathway involving WRKY10, miR397a, and LAC2 in the control of seed size in *Arabidopsis*. These findings expand our understanding of the complex regulatory network governing seed development and provide new targets for the improvement in seed traits in crops. Future research should focus on elucidating the biochemical functions of the miR397-LAC module in endosperm development, investigating the conservation of this module across diverse plant species, and exploring the potential applications of this knowledge in crop improvement. By integrating insights from basic plant biology with applied research, we can develop innovative strategies to enhance seed size and yield, ultimately contributing to global food security.

## Data Availability

The original contributions presented in the study are included in the article, further inquiries can be directed to the corresponding author.
